# Supplementary material for: A D-alanine aminotransferase S180F substitution confers resistance to β-chloro-D-alanine in Staphylococcus aureus via antibiotic inactivation
Source: bioRxiv. 2025 Aug 18:2025.08.17.668425. Preprint. [Version 1] doi: 10.1101/2025.08.17.668425 (PMC12393368; doi:10.1101/2025.08.17.668425)
Supplement: Supplement 2 [file media-2.pdf]

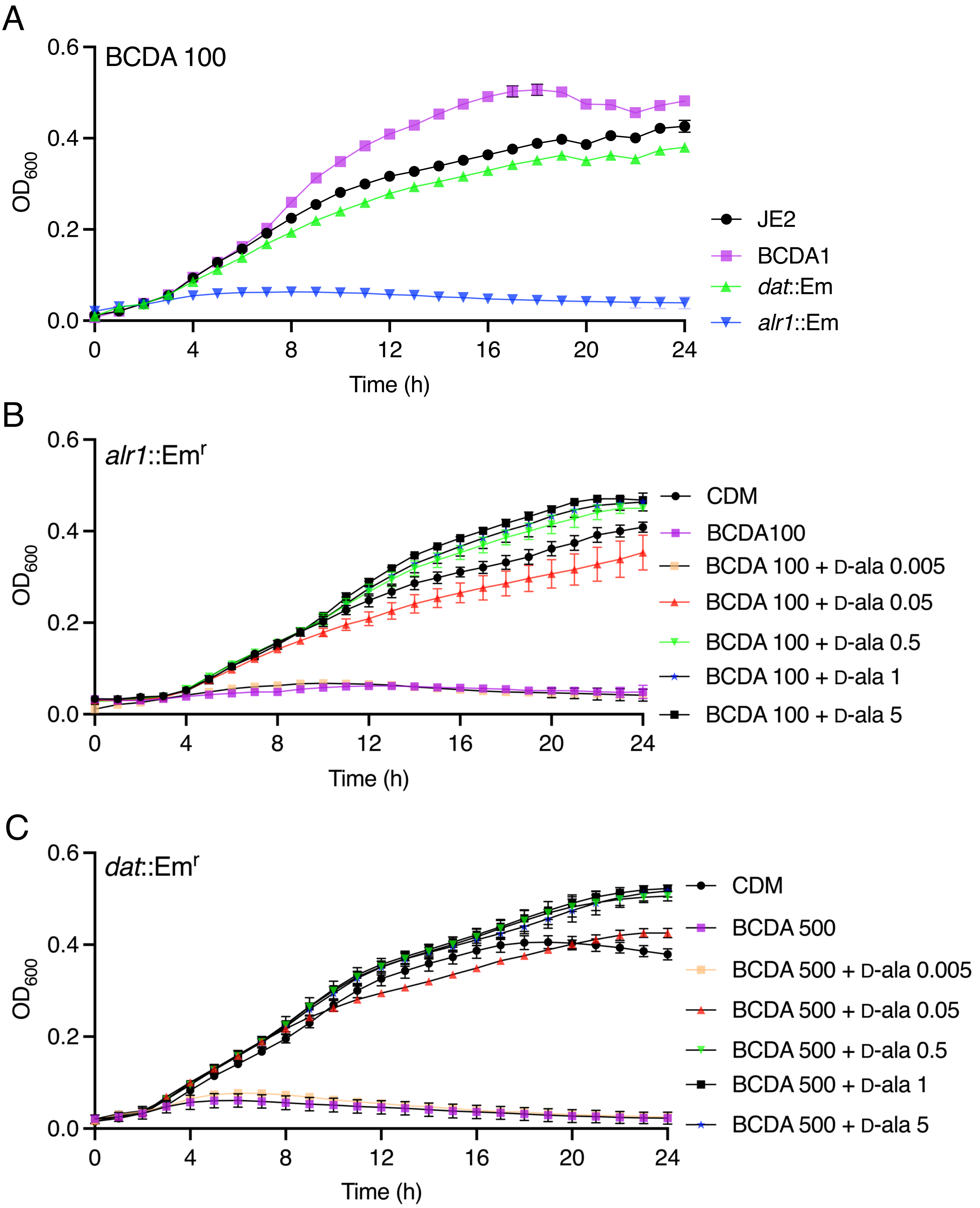


**Fig. S2. Exogenous D-alanine restores growth of *alr1* and *dat*::Em$^r$ mutants inhibited by BCDA. A.** Comparison of JE2, BCDA1, NE1305 (*dat*::Em) and *alr1* growth in CDM supplemented with BCDA 100 µg/ml. **B.** Comparison of *alr1* growth in CDM supplemented with BCDA 100 µg/ml alone or with exogenous D-alanine concentrations from 0.005 to 5 mM. **C.** Comparison of *dat*::Em$^r$ growth in CDM supplemented with BCDA 100 µg/ml alone or with exogenous D-alanine concentrations from 0.005 to 5 mM. The data presented are the average of at least 3 biological replicates and standard deviations are shown.
